# The MassBank contributions of the mFam collaboration

**DOI:** 10.1007/s11306-026-02480-y

**Published:** 2026-07-02

**Authors:** Anusha Ahlendorf, Asaph Aharoni, Khabat Vahabi, Tillmann G. Fischer, Pierre-Marie Allard, Megan Augustin, Ulschan Bathe, David I. Broadhurst, Corey Broeckling, Joerg Buescher, Adrian Covaci, Katyeny Manuela da Silva, Ric C. H. de Vos, Micha Gracianna Devi, Stefanie Döll, Maximilian Frey, Andrej Frolov, Emmanuel Gaquerel, Vasuk Gautam, Esteban Charria-Girón, Alain Goossens, Jeremy Grosjean, Maria Halabalaki, Elias Iturrospe, Kim Kultima, Stephanie Herman, Toni M. Kutchan, Romain Larbat, René Meier, Eleni V. Mikropoulou, Gregory Mouille, Luca Nicolotti, Nir Shahaf, François Perreau, Pierre Pétriacq, Michael Reichelt, Stacey N. Reinke, Rani Robeyns, Alena Soboleva, Otmar Spring, Akshai Parakkal Sreenivasan, Alain Tissier, Jean Chrisologue Totozafy, Hiroshi Tsugawa, Josep Valls-Fonayet, Maria van de Lavoir, Justin J. J. van der Hooft, Fredd Vergara, David Wishart, Ludger A. Wessjohann, Jean-Luc Wolfender, Jörg Ziegler, Gerd Ulrich Balcke, Steffen Neumann

**Affiliations:** 1 UKJ Data Integration Center, Stoystrasse 3, Jena, Germany; 2 Leibniz Institute of Plant Biochemistry, Weinberg 3, D-06120 Halle/S, Germany; 3 Department of Plant and Environmental Sciences, Weizmann Institute of Science, Rehovot, Israel; 4 Leibniz Institute of Vegetable and Ornamental Crops (IGZ) e.V, Großbeeren, Germany; 5 Faculty of Chemistry, Leipzig University, Johannisallee 29, 04103 Leipzig, Germany; 6 University of Fribourg, Chemin du Musée 10, CH-1700 Fribourg, Switzerland; 7 Swiss Institute of Bioinformatics, Lausanne, Switzerland; 8 Donald Danforth Plant Science Center, 975 North Warson Rd, St. Louis, MO USA; 9 Biotechnology of Horticultural Crops, Technical University of Munich, Liesel-Beckmann- Straße 1, 85354 Freising, Germany; 10 School of Science, Edith Cowan University, Joondalup, Western Australia Australia; 11 Colorado State University, C-115 Microbiology Building, 2021 Campus Delivery, Fort Collins, CO 80523 USA; 12 Max Planck Institute of Immunobiology and Epigenetics, Stübeweg 51, 79108 Freiburg, Germany; 13 Toxicological Centre, University of Antwerp, Universiteitsplein 1, 2610 Antwerpen, Belgium; 14 Bioscience, Wageningen Plant Research, Wageningen University & Research, 6708 PB Wageningen, The Netherlands; 15 German Centre for Integrative Biodiversity Research (iDiv) Halle-Jena-Leipzig, Leipzig, Germany; 16 Institute of Biodiversity, Friedrich Schiller University, Jena, Germany; 17 Biochemistry Center, Heidelberg University, 69120 Heidelberg, Germany; 18 Institut de Biologie Moléculaire des Plantes du CNRS, 12 Rue du Général-Zimmer, Strasbourg, 67000 France; 19 Norton Neuroscience Institute, Norton Healthcare, Louisville, KY USA; 20 Bioinformatics Group, Wageningen University & Research, 6708 PB Wageningen, The Netherlands; 21 Department of Plant Biotechnology and Bioinformatics, Ghent University, 9052 Ghent, Belgium; 22 VIB Center for Plant Systems Biology, 9052 Ghent, Belgium; 23 Department of Botany and Zoology, Stellenbosch University, Matieland, 7600 South Africa; 24 Université de Lorraine, INRAEUMR1121 LAE, Nancy, France; 25 Division of Pharmacognosy and Natural Products Chemistry, Department of Pharmacy, National and Kapodistrian University of Athens, Panepistimiopolis Zografou, 15771 Athens, Greece; 26 Department of Medical Sciences, Uppsala University, 75185 Uppsala, Sweden; 27 Institut Agro, Université d’Angers, INRAE, IRHS, SFR QUASAV, 49000 Angers, France; 28 Université Paris-Saclay, INRAE, AgroParisTech, Institute Jean-Pierre Bourgin for Plant Sciences (IJPB), Versailles, 78000 France; 29 The Australian Wine Research Institute, Metabolomics Australia, PO Box 46, Glenside, SA 5065 Australia; 30 Univ. Bordeaux, INRAE, UMR1332 BFP, 33882, Villenave d’Ornon, France; 31 Max Planck Institute for Chemical Ecology, Hans-Knöll-Straße 8, 07745 Jena, Germany; 32 Department of Biology, University of Hohenheim, Garbenstr. 30, 70599 Stuttgart, Germany; 33 Department of Cell and Metabolic Biology, Leibniz Institute of Plant Biochemistry, 06120 Halle (Saale), Germany; 34 Institute of Pharmacy, Martin-Luther University Halle-Wittenberg, 06120 Halle (Saale), Germany; 35 Department of Biotechnology and Life Science, Tokyo University of Agriculture and Technology, 2-24-16 Nakamachi, Koganei, Tokyo, 184-8588 Japan; 36 INRAE, UMR1366 Oenology, Univ. Bordeaux, 33880 Villenave d’Ornon, France; 37 Department of Biological Sciences, University of Alberta, Edmonton, AB T6G 2E9 Canada; 38 Institute of Chemistry, Martin-Luther-Universität Halle-Wittenberg, Halle (Saale), Germany; 39 Institute of Pharmaceutical Sciences of Western Switzerland, University of Geneva, Geneva, Switzerland; 40 School of Pharmaceutical Sciences, University of Geneva, Geneva, Switzerland

**Keywords:** Metabolomics, Tandem mass spectrometry, Reference spectra, Spectral libraries, Data processing, FAIR data, Open science

## Abstract

**Introduction:**

The analysis of metabolic profiles using high resolution mass spectrometry (MS) data provides deep insights into biological processes. In metabolomics, MS analysis generates a large number of features that represent metabolites. However, identifying specific metabolites from these features can be challenging. One of the major bottlenecks in the metabolomics field is the identification of MS features, which is a prerequisite for any biochemical interpretation. By identifying similarities and differences within a metabolite family (mFam), evaluating MS features at the metabolite family level can help assigning functional roles to individual MS features. These data can help interpreting metabolic pathways and processes within a biological system. For the assignment of metabolite families to MS features, it is important to have good quality, reliable, and comprehensive spectral libraries.

**Objective:**

We initiated a global effort to collect high-resolution MS/MS spectra of metabolites from labs working in different fields, including metabolomics of animals, microorganisms, and plants. The mFam-MS/MS collection delivers valuable training data to assign machine-readable classified information on the unknown metabolites.

**Results:**

The mFam collaboration used a standardized metadata template and has developed a globally curated MS/MS spectral library of 7,872 spectra with 2,126 unique metabolites. This library was compiled from 47 datasets contributed by 25 laboratories measured on 12 instrument types, including QTOF, Orbitrap, and Ion Mobility-QTOF systems. It comprises 4,646 spectra in positive mode and 3,226 in negative mode. This standardized resource significantly enhances metabolite identification capabilities, supports the development of machine learning-based annotation tools, and accelerates the discovery of novel metabolites. All spectra are available under the collective contributor label mFam in the MassBank system, including the web interface and the 2025.10 data release available at GitHub and Zenodo.

**Supplementary Information:**

The online version contains supplementary material available at https://doi.org/10.1007/s11306-026-02480-y.

## Introduction

Mass spectrometry is an important analytical chemistry method for the detection, quantification, and identification of small molecules with several applications in diverse fields. These include but are not limited to toxicology (Garg & Zhang, 2016; Van Wijk et al., 2019), exposomics (Travis et al., 2021), environmental research (Alseekh et al., 2021) and metabolomics in the life sciences and biomedical research (Bauermeister et al., 2022). In case of untargeted strategies in these fields, one of the main bottlenecks is the annotation and characterization of molecular structures, which is not readily available from the spectral data.

In addition to the chromatographic retention time and the accurate mass, the fragmentation pattern in tandem mass spectrometry (MS/MS) can carry structural information about detected molecules. Databases of fragmentation spectra, also known as spectral libraries, contain MS/MS spectra of compounds with known identity. Comparison of an unidentified spectrum against the spectral library provides information toward its identification.

Several spectral libraries exist, both commercial and open access. These libraries and the overlap in their content have been reviewed (Bittremieux et al., 2022; Kind et al., 2018; Vinaixa et al., 2016). Open and FAIR (Wilkinson et al., 2016) spectral libraries provided by the research community include MassBank (Horai et al., 2010; Neumann et al., 2026), MoNA (*MassBank of North America*, n.d.), HMDB (Wishart et al., 2022) and GNPS libraries (Wang et al., 2016). The content in these databases can also be compared to the set of known metabolites in organism-specific pathway databases (Frainay et al., 2018). Efforts to enhance the accessibility and reusability of mass spectral library data, following FAIR principles, have been further explored in recent studies, such as the development of a matchms-based (Huber et al., 2020) spectral library cleaning and metadata-harmonization pipeline (de Jonge et al., 2024).

The power of metabolite identification strongly depends on the quality of data. The MS/MS spectra can vary depending on the instrument, collision energy, mass resolution, fragmentation mode, and many other factors and hence the spectral libraries can be improved with spectral data from as many measurement conditions as possible.

The creation of spectral libraries typically begins with obtaining and preparation of authentic reference compounds, progressing to data acquisition, data processing and finally generation of the spectral library itself. In two studies (da Silva et al., 2022; Stein, 2012), the authors discuss measures to assess and improve library quality. Several software programs and packages facilitating the generation of spectral libraries are currently available. For instance, the RMassBank package (Stravs et al. 2017; Stravs et al., 2013) provides functions for processing spectral data of reference compounds, including a batch-wise mass recalibration after annotation of molecular formulas to fragments.

The Weizmass library (Shahaf et al., 2016) was created from data-independent acquisition (DIA) all-ion measurements, and processed with a custom pipeline written in R. RAMClustR (Broeckling et al., 2014), also written in R, was designed specifically for the processing of all-ion fragmentation data in DIA mode. The Curatr application provides a graphical user interface (GUI) for the acquisition, processing and curation of spectral libraries (Palmer et al., 2018). Tsugawa et al. created the MS-DIAL software (Tsugawa et al., 2015) for processing LC-MS raw data, including both data-dependent acquisition (DDA) and DIA and processing. Bruderer et al. (Bruderer et al., 2018) also described a workflow for creating an Accurate Mass Metabolite Library (AMML) for SWATH data from flow-injection (FIA) measurements. Complementing these efforts, Brungs et al. introduced MSⁿLib (Brungs et al., 2025), a large-scale open spectral library built with a high-throughput flow-injection DDA pipeline on an Orbitrap ID-X. Unlike MS2 only libraries, MSⁿLib acquires multi-stage fragmentation trees by iteratively fragmenting product ions, generating ~ 2.3 million spectra (including > 357,000 MS² spectra) across 30,008 unique small molecules. The pipeline integrates automated metadata curation, quality control, and spectral merging in mzmine, expanding open MSⁿ resources for compound annotation and machine learning. The survey by Frainay et al. (Frainay et al., 2018) showed that less than 5% of known metabolites from pathway databases have experimental MS/MS spectra. Interpretation of the remaining 95% is supported by metabolite family-aware annotation tools, which in turn need at least some coverage of the metabolite family with reference spectra for machine learning approaches such as implemented in, e.g., MS-Finder (Lai et al., 2018; Tsugawa et al., 2019), MetFamily (Treutler et al., 2016), or SIRIUS (Dührkop et al., 2021; Hoffmann et al., 2022; Stravs et al., 2022). An example for monoterpene indole alkaloids was shown in (Szwarc et al., 2025).

**Fig. 1:**
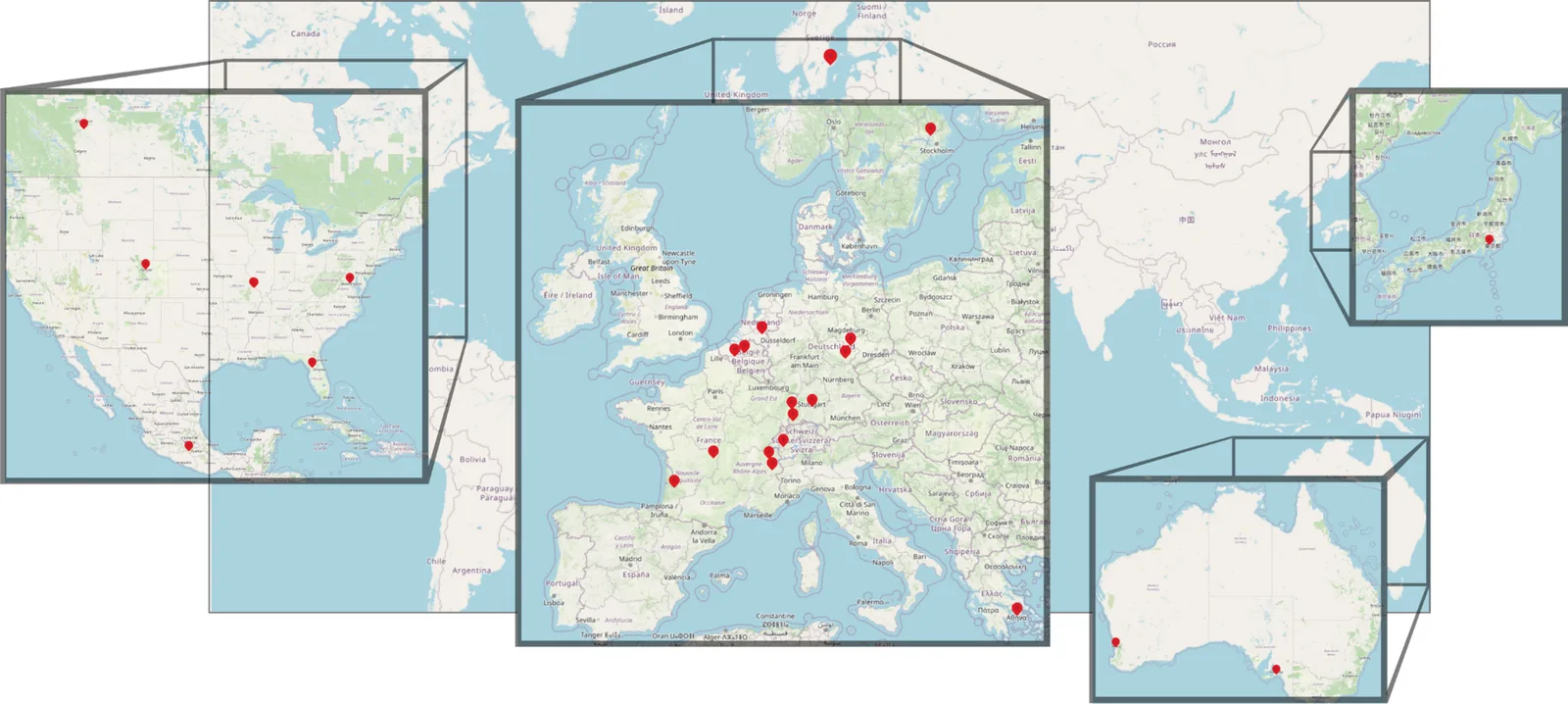
Global distribution of the 25 laboratories contributing spectral library data in the mFam efforts

To address this gap, we coordinated a global effort to gather high-resolution MS/MS spectra from laboratories working on a wide range of different biological systems, including animals, microorganisms, and plants. This collaborative effort involved 25 laboratories (see Fig. 1), which collectively contributed 47 different datasets. Various analytical platforms were utilized, such as different Thermo Orbitrap models, or QTOF instruments from Agilent, Bruker, Sciex and Waters. In different laboratories, data were collected using both DDA and DIA acquisition modes, covering both positive and negative ionization methods.

This paper presents the approaches, tools, and key lessons learned during the creation of a spectral MS/MS library that is now included in MassBank. It describes the data acquisition, processing, and curation processes. Additionally, we share insights into overcoming challenges related to data standardization, quality control, and the implementation of FAIR data principles. Our approach aims to improve the utility of spectral libraries and lay a solid foundation for future advances in metabolite annotation and structural characterization.

## Methods

We approached different laboratories working in metabolomics, asking for their in-house spectral data to be published in MassBank. Together, all partners comprise the mFam collaboration. The global distribution of contributors is shown in Fig. 1. Rather than requesting newly acquired data for inclusion in MassBank, we accepted spectral data directly from LC–MS raw data files, provided either in vendor-specific formats or in mzML. In some cases, contributors had already processed the in-house spectral libraries tailored for metabolite identification with the used instruments, and subsequently, we included those libraries.

Since the MassBank records carry a rich set of metadata and analytical information, record creation is not a trivial task. We provided contributors with a standardized spreadsheet template to enter the analytical metadata, instrument settings and data acquisition strategy (either DDA or DIA) on the one hand, and chemical information about the reference compounds and their analytical information (e.g., adduct-ion information, the expected retention time on the used analytical setup) on the other.

Due to the heterogeneous nature of the provided data, we created several processing modules to calculate or infer missing information. One aim was to keep the manual effort minimal. It is sufficient if one of the chemical identifiers like SMILES, InChI or a PubChem CID is correctly provided, and the others can be obtained through identifier conversion and translation methods. This was particularly important as contributors come from different research settings, each using a variety of instrumentation and data formats.

We specifically designed the workflow so that only one type of chemical identifier—such as SMILES, InChI, or a PubChem CID—is needed to link each compound’s structure with its corresponding metadata. Using these identifiers, the system automatically derived key chemical attributes such as molecular formula, monoisotopic mass, and structural information, ensuring consistency while minimizing manual effort and simplifying data sharing. A template spreadsheet for metadata collection with several example entries is provided as Supplemental File S1. To avoid missing, partial, or incorrect information, several checks are performed upfront:

The spreadsheet contains information about the author and additional provenance (e.g., creation date and optionally a publication describing the spectral data) and information for the spectral data. Another part contains information about the instrumentation, and several analytical settings about chromatography and mass spectrometry. The chemical information in the metadata spreadsheet is needed for two reasons. First, it allows linking out to other chemical databases with additional information not available in MassBank. Second, in our pipeline, the information is used to identify the MS/MS spectrum of a compound among all MS/MS spectra in a raw data file, e.g. from LC-MS/MS data.

If the PubChem compound CID was provided, we retrieved the SMILES, InChI and InChIkey from PubChem via the PubChem REST API. In case contributors also provided SMILES and/or InChI in their compound metadata, we checked that they matched the structures from the upstream database, to raise a warning otherwise. If instead a structure was provided (as SMILES or InChI), the PubChem database was queried using the webchem package to retrieve the database identifier. Furthermore, the pipeline derives the molecular formula and accurate mass directly from the provided chemical identifiers, thereby avoiding conflict or ambiguity. There have been cases where contributors have provided inconsistent information, with different stereochemistry or even isomers. We validated the consistency among the provided and automatically converted structural information to find these cases and raise an error, so they could review and update their submissions.

Within the mFam collaboration, contributors have used different data and file formats to provide the MS/MS data. The majority of contributors provided the raw instrument data of the measured metabolite standards. These came either in the respective vendor formats, or already converted to open file formats like mzML (and mzXML in one case). Such data was processed with MS-DIAL for feature detection and precursor mass assignment, resulting in an MSP file with all MS/MS spectra (Tsugawa et al., 2015). Several contributors provided already processed spectral files, again some in vendor formats (e.g., Bruker .library) and several in open text formats like MGF or MSP. In these cases the MS-DIAL processing was not required.

The metadata spreadsheet links raw LC-MS data files, the associated MS/MS library, and the MS/MS acquisition mode. To extract spectra for MassBank records, precursor *m/z* values were derived from known adducts or, if unavailable, estimated using common ionization species and adducts. Matching MS/MS spectra were filtered by retention time and by instrument-specific mass error.

Since the target compound is not necessarily the most abundant peak in the chromatogram, extraction of the MS/MS spectra benefits from the provided retention time given in the metadata sheet. In case of multiple matching MS/MS spectra for a precursor *m/z*, the pipeline extracts the best matching MS/MS spectrum for a compound, using a weighted score as heuristic that incorporates the target retention time and richness (i.e., number of peaks and their average intensity) in the MS/MS spectrum. Spectra were selected using a retention time tolerance of 0.2 min and a precursor *m/z* tolerance of 25 ppm, matching the observed precursor ion against the expected adduct provided in the contributor’s metadata. When multiple candidate spectra were present within this tolerance range, we prioritized spectra with the highest base-peak intensity and the maximum number of fragment ions that passed the RMassBank noise-to-signal filters. For Flow Injection Analysis (FIA) the precursor *m/z* was matched within a 10 ppm tolerance.

Finally, the metadata, chemical, and spectral information are formatted into the MassBank record format using the RMassBank package. We have extended the RMassBank Package to retrieve the ChemOnt classification and include this in the MassBank records. Following the semi-automated spectra extraction and data enrichment, the individual libraries were returned to the respective contributors of the mFam consortium for manual curation.

For the chemoinformatic analysis, SMILES strings for all mFam entries and those in MassBank release 2025.05.01 were canonicalized using RDKit, and invalid structures were excluded. Extended-connectivity fingerprints (ECFP4; Morgan radius 2, 4096 bits) were computed and used for the molecular representation. Chemical space was visualized by projecting the combined fingerprint matrix onto a two-dimensional UMAP. Structural novelty for each mFam entry was quantified as the maximum Tanimoto similarity to any compound in the MassBank baseline, computed by exhaustive pairwise search using RDKit bulk Tanimoto routines (Compounds with similarity ≥ 0.99 were excluded). For Fig. 3a, the ten most structurally distinct compounds per contributing lab were identified by sorting deduplicated mFam entries by ascending maximum Tanimoto similarity and retaining the top-10 per lab. Labs were ranked by the mean maximum Tanimoto similarity of their respective top-10 most novel compounds, and the four most novel structures from the five highest-ranking labs were depicted in Fig. 3c. All analyses were performed in Python using RDKit, pandas, NumPy, and UMAP-learn, and are fully reproducible via the Jupyter Notebook available at github.com/ECharria/mFam-contributions.

## Results

The contributions provided a unique insight into the breadth of in-house libraries found in participating metabolomics laboratories across the world. This begins by deciding how to run the reference compounds on the analytical setup.

*Injection of one chemical reference compound into an LC/MS(/MS) method.* Here, the reference compound is injected just like a normal sample, using the established analytical setup in the laboratory. This approach was used in 27 datasets. A benefit of this approach is that the reference compound can be expected to be easily identifiable with matching mass and retention time.

A variant to the previous approach is to *combine a number of compounds into a mixture of the reference compounds.* Here, some optimization was required to avoid co-elution of the substances. This approach was used in 20 datasets. The extraction of the reference spectra was slightly more complex, but the multiplexing of the measurement drastically reduces the required instrument time.

*With flow injection analysis (FIA)*, even more measurement time reduction is possible. Due to the lack of chromatography, only individual compounds can be injected, and the measured data lacks the retention time information. The FIA strategy was used in 9 datasets.

*The most common data acquisition approach is to use DDA acquisition (38 contributors)*, where the isolation window has to be specified, or DDA is chosen, possibly with an inclusion list. Ten contributors have created spectral libraries with DIA acquisition strategies, e.g. called MS^E^ for Waters or All Ion Fragmentation (AIF) for Agilent instruments, while one contributor used both DDA and DIA approaches. The benefit is that there is no need to specify which precursor or adduct to include for each authentic standard. The downside is the requirement to deconvolute the fragmentation spectra to obtain virtual pure MS/MS spectra.

Finally, the contributions differed in the amount of data preprocessing performed by the contributors. If possible, we asked for the raw data to apply the same data processing workflow, and applied the workflow described in the methods section above. However, pre-processed datasets provided by contributors required additional customization to standardize their diverse formats. For instance, Stacey Reinke contributed untargeted LC-MS/MS data (Naz et al., 2017), where metabolomic analysis was performed on biological samples, and a library of reference spectra was collected via FIA of individual matrix-free chemical standards. Corey Broeckling provided Waters MS^E^ data processed with RAMClustR (Broeckling et al., 2014), which organizes data into clusters of deconvoluted MS/MS spectra in a spectral library. Stefanie Döll supplied spectral files generated using Bruker software. These varied formats—including Excel spreadsheets with different layouts, MGF files, and vendor-specific exports—were parsed and standardized using custom scripts, ensuring consistent spectral quality while respecting the unique workflows of each contributor.

We also compiled which instruments and instrument types were used by the mFam consortium. The most frequent instrument types used for measurements in the database are ESI-QTOF (29), and APCI or ESI with Orbitrap-MS (18). The instruments used for measurements in the database are Bruker MicrOTOF-Q, Impact II, maXis impact HD and timsTOF, Sciex TripleToF 5500 and 5600, Agilent 6530 and 6560, Waters Xevo G2 and Synapt, and several Thermo Scientific Orbitrap instruments (XL and Elite, Q-Exactive Focus and Plus). Only mass spectrometry data with MS2 were added to MassBank.

The majority of spectra (59%) have been measured in positive mode, and 98% of spectra have been measured using ESI ionization. In the content tab of the MassBank interface, it is possible to filter for the mFam Contributor (and further filter criteria) to obtain record counts. The most frequent ionization species used for spectra in this database are [M + H]^+^, [M-H]^−^, and [M+NH_4_]^+^. While MassBank records do not capture the chromatographic setup in detail required for retention time modelling, different chromatographic systems and gradients have been used and a preference is evident: 93% of measurements were done on an Acquity CSH C18 column, and 82% of compounds in the collection were measured at retention times below 15 min.

**Table 1:** Total number of spectra and unique compounds. Some compounds were measured in both positive and negative ionization mode

	Positive ionization	Negative ionization	Total
Total no of spectra	4646	3226	7872
Unique Compounds	1757	1030	2126

**Fig. 2:**
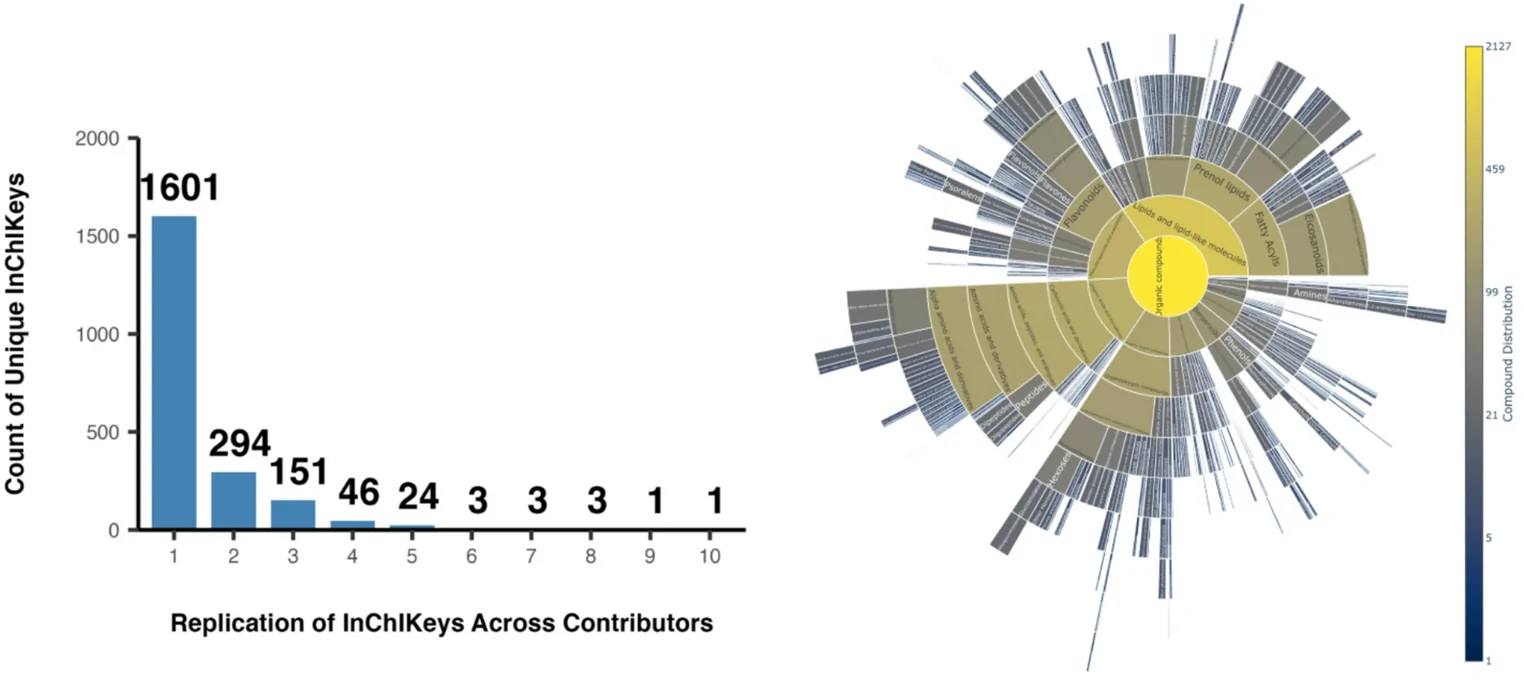
Left: The vast majority of compounds (1601) were unique among the mFam contributions and measured by a single contributor. There are also in total 526 cases where compounds are measured on different instrumentation, which also allows to compare spectra across analytical setups. Right: Distribution of the compound classes of the metabolites in the mFam dataset (according to the ChemOnt classification), where the dominant superclasses are phenylpropanoids, polyketides, lipids, terpenoids and organoheterocyclic compounds. The Sunburst plot provides the compound classes at increasing levels of detail, and the relative (angle covered) and absolute (colour code) number of compounds in each respective class. Note that not all compounds are classified down to the most detailed level. The interactive HTML version of this plot can be found at *Sunburst-mFam.html* and in the supplemental material

After the preprocessing and QC steps, the mFam spectral data collection contains 7872 spectra, originating from 2126 compounds with unique structures, see Table 1. These numbers and additional information about instrument types or the interactive compound class sunburst plot are also available from the MassBank contents page when filtering for the “mFam” contributor. We also checked how common compounds are, i.e. how often they were measured by different contributors, see Fig. 2 (left). The five most common compounds are *Caffeoyl quinic acid*, *Quercetin 3-rutinoside*, *Naringenin*, *Sinapic acid*, and *Luteolin*. The compound class distribution according to the ChemOnt (Fig. 2, right) is enriched by phenylpropanoids and polyketides initially and also in lower level by lipids and terpenoids which reflects the major plant science focus of the contributing laboratories to this study and consistent with prevalence of the phenolic compounds among the more common compounds.

To demonstrate the chemical coverage of the added compounds, we used the Classyfire tool (Djoumbou Feunang et al., 2016) to assign the compound classes and show their distribution across mFam entries in Fig. 2 (right).

**Fig. 3:**
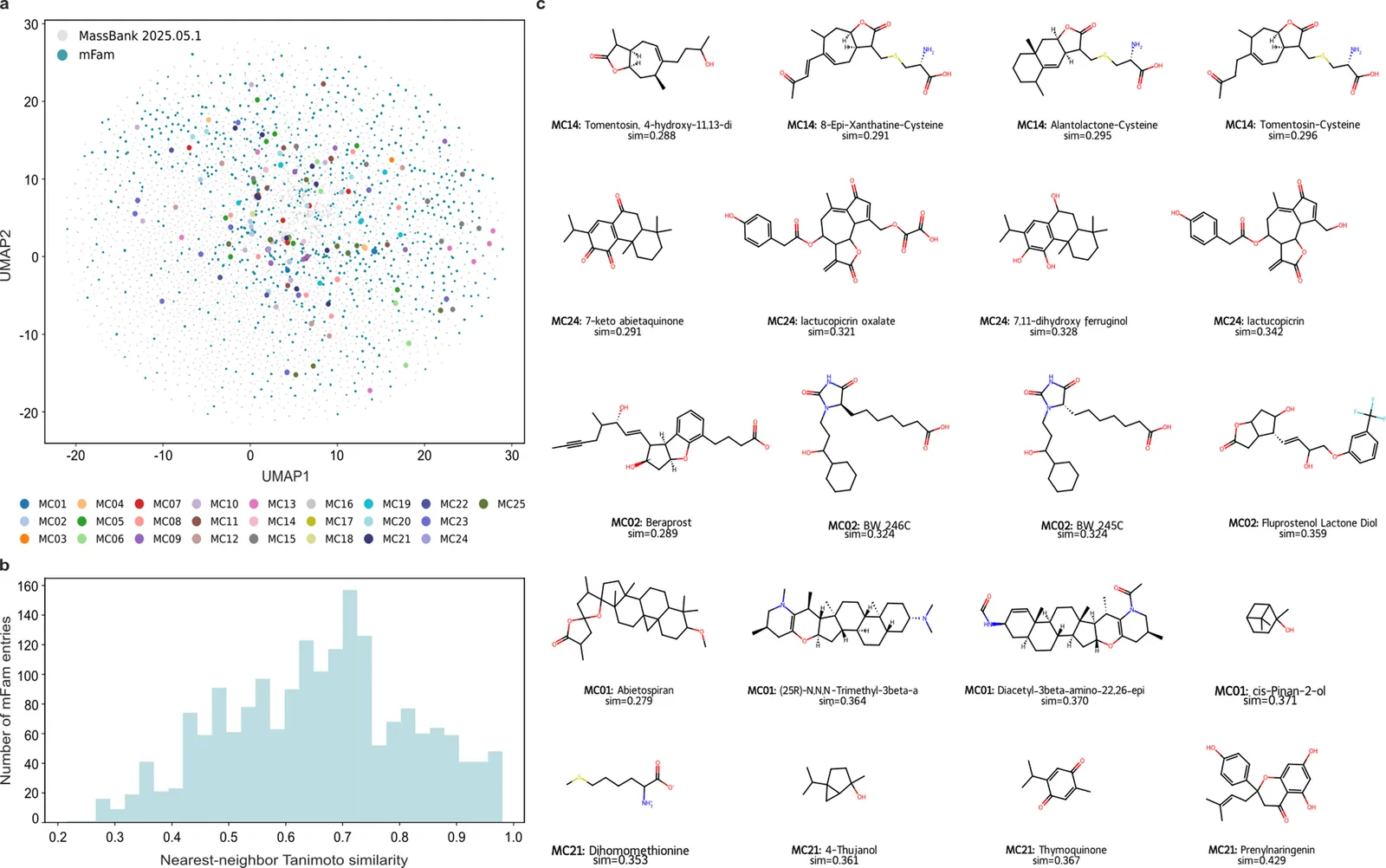
**a** UMAP projection of ECFP4 fingerprints showing the chemical space covered by MassBank release 2025.05.1 (grey) and mFam entries (blue). The top-10 most structurally novel compounds from each contributing lab are highlighted and colored by contributor identifier. **b** Distribution of nearest-neighbor Tanimoto similarities between mFam compounds and their closest structural match in MassBank release 2025.05.1 after excluding near-identical matches (similarity ≥ 0.99), highlighting the structural novelty of the non-redundant mFam contributions. **c** Chemical structures of the top-4 most structurally distinct compounds from the top-5 most novel labs (MC14, MC24, MC02, MC01, and MC21), ranked by the mean nearest-neighbor Tanimoto similarity of their top-10 highlighted entries shown in panel a. Figure created with the Jupyter Notebook available from *github.com/ECharria/mFam-contributions*

Beyond the diversity of contributions and compound classes, an important question is how these new entries expand the chemical space represented in MassBank. To address this, we compared the chemical space covered by mFam entries with those in the MassBank release 2025.05.1 using Extended-connectivity fingerprints (ECFP4) and dimensionality reduction (Fig. 3). This analysis allows us to assess both the overall coverage of chemical space and the structural novelty of the newly contributed compounds by the mFam efforts. Excluding near identical matches ( > = 0.99 tanimoto similarity) to existing MassBank entries, 1879 new entries were added, which exhibited a median similarity index of 0.667 among them (Fig. 3b). To highlight the coverage per contributor, the ten most structurally distinct compounds from the 25 participating labs are highlighted in Fig. 3a. The five labs whose top-10 most novel compounds showed the lowest mean nearest-neighbor similarity to MassBank entries are shown in Fig. 3c. For example, 6 of the 20 most structurally distinct mFam compounds are sesquiterpenes of various backbone types such as eudesmanolides and xanthanolides, that are characteristic for plants of the Asteraceae family (Frey et al., 2024), and their Michael adducts (Frey, 2020).

## Discussion

During the mFam activities, the biggest challenge faced was the chemical data management. Each contributor knows their in-house spectral library best, and also the metabolites that are their usual suspects. We requested the metadata from each contributor in a specific tabular form, containing sufficient metadata to create MassBank records. Here, we found that this manual transfer process had the largest opportunity to introduce errors. The development of automated preprocessing pipelines reduced manual curation by systematically checking for missing metadata, filename consistency, and retention time information, demonstrating the value of quality control automation in handling diverse contributor formats. We were able to identify issues, and iterated with contributors to achieve consistency. Once the metadata was in shape, the above-described workflow using MS-DIAL for preparing the MS/MS spectra, and extracting the target compound MS/MS spectrum was rather straightforward. Our analysis of spectral library creation workflows within the mFam collaboration reveals that, despite diverse data acquisition methods, standardization is achievable through robust metadata management and preprocessing workflows. Chemical structure redundancy, achieved by providing multiple identifiers (InChI, SMILES, PubChem CID), proved essential for cross-validation, improving data quality and consistency. Together, these contribute to the FAIRness of the mFam contribution. Challenges persist in managing vendor-specific data formats and heterogeneous preprocessing practices. For example, we initially observed some raw data where the acquisition properties reported centroidization, while the spectra did not appear to be centroided. Future efforts should prioritize improving interoperability among MS processing platforms and establishing standardized quality metrics, paving the way for broader adoption of these workflows in metabolomics research.

During the collection of the mFam spectral data, we did not prescribe which compounds and spectra should be submitted. This means on the one hand that 88% of the mFam compounds are new to MassBank and can thus extend the training data of upcoming machine learning methods, while the different research backgrounds of the labs contributed to the diversity of the novel structures. On the other hand, nearly 25% of the mFam compounds were measured on more than one analytical setup, which increases the chance that a laboratory running some instrument can find comparable reference spectra in MassBank. This replication of spectra on different analytical setups can also improve the robustness of machine learning methods.

Our structural comparison of individual mFAM contributions revealed only moderate Tanimoto similarity to known structural relatives in MassBank release 2025.05.1. We therefore conclude that metabolites contributed by different laboratories—including monoterpenes, terpene lactones, diterpenoids, azoles and azolidines, steroidal diterpenes, coumarans, phenols, and quinones—help fill structural gaps across the broader metabolic space.

## Conclusion

The mFam-MS/MS Collection in MassBank is a valuable and freely available resource for the metabolomics community. The spectral data collection can be used to identify metabolites in a variety of biological samples, including plants, animals, and microorganisms. It can also be used to study the metabolism of drugs and natural products. An area that could be improved is the diversity of the collection. The collection contains a wide variety of spectra, many of which are found in the plant kingdom, while the collection does not contain many spectra from aquatic organisms.

A central motivation of the mFam collaboration was to leverage the collective expertise of several labs in the global metabolomics community. Individual laboratories often maintain rich but isolated in-house spectral libraries, and pooling these resources enables addressing gaps in chemical coverage more efficiently than any single group could achieve. The mFam collaboration has provided valuable insights into the characteristics of the datasets obtained, including the number of datasets that use DDA, DIA or FIA strategies, comparison across TOF or Orbitrap instrumentation, and other characteristics. The experience of processing MS/MS data from a range of instruments and laboratories has highlighted the importance of metadata, unique identifiers, checks for consistency of identifiers, adduct availability, and the number of peaks. The resulting resource not only increases coverage in MassBank but also strengthens interoperability and reproducibility across metabolomics laboratories worldwide.

By adding 7872 mass fragmentation spectra from 2126 unique compounds, of which 1879 are new to MassBank, the mFam collaboration has become the 5th largest contributor in MassBank. The creation of diverse and reliable spectral libraries will continue to be crucial for the identification of MS features and machine learning tasks.

## Supplementary Information

Below is the link to the electronic supplementary material.

Supplementary Material 1

## Data Availability

The records described in this manuscript have been incorporated into the GitHub MassBank-data repository with commit [a91b1ca] (https://github.com/MassBank/MassBank-data/commit/a91b1ca4841aea536545f7c1d452c1f80d225e84) . Along with other MassBank-data contributions, the mFam contribution has been released as [Release version 2025.10](https:/github.com/MassBank/MassBank-data/releases/tag/2025.10) , and deposited to Zenodo as (MassBank consortium and its contributors, 2025). It is also available from the Software Heritage archive under [swh:1:dir:0332839b153f8d587c4e140d1f9c6ea048ad56d1] (https://archive.softwareheritage.org/swh:1:dir:0332839b153f8d587c4e140d1f9c6ea048ad56d1) .
